# Impact of semaglutide on high-sensitivity C-reactive protein: exploratory patient-level analyses of SUSTAIN and PIONEER randomized clinical trials

**DOI:** 10.1186/s12933-022-01585-7

**Published:** 2022-09-02

**Authors:** Ofri Mosenzon, Matthew S. Capehorn, Alessandra De Remigis, Søren Rasmussen, Petra Weimers, Julio Rosenstock

**Affiliations:** 1 Diabetes Unit, Department of Endocrinology and Metabolism, Hadassah Medical Center, Faculty of Medicine, Hebrew University of Jerusalem, PO Box 12000, Jerusalem, Israel; 2 Clifton Medical Centre, Rotherham Institute for Obesity, Rotherham, UK; 3 Novo Nordisk A/S, Søborg, Denmark; 4 Velocity Clinical Research at Medical City, Dallas, TX USA

**Keywords:** Semaglutide, GLP-1RAs, High-sensitivity C-reactive protein, Inflammation, HbA_1c_, Body weight, SUSTAIN, PIONEER, Type 2 diabetes, Chronic kidney disease

## Abstract

**Background:**

Exploratory analysis to determine the effect of semaglutide versus comparators on high-sensitivity C-reactive protein (hsCRP) in subjects with type 2 diabetes.

**Methods:**

Trials of once-weekly subcutaneous (SUSTAIN 3) and once-daily oral (PIONEER 1, 2, 5) semaglutide with hsCRP data were analyzed. Subjects with type 2 diabetes (N = 2482) received semaglutide (n = 1328) or comparators (placebo, n = 339; exenatide extended-release, n = 405; empagliflozin, n = 410). hsCRP ratio to baseline at end-of-treatment was analyzed overall, by clinical cutoff (< 1.0, ≥ 1.0 to ≤ 3.0, or > 3.0 mg/L), by tertile, and by estimated glomerular filtration rate in PIONEER 5 (a trial which was conducted in a population with type 2 diabetes and chronic kidney disease [CKD]). Mediation analyses assessed the effect of change in glycated hemoglobin (HbA_1c_) and/or change in body weight (BW) on hsCRP reductions.

**Results:**

Geometric mean baseline hsCRP was similar across trials (range 2.7–3.0 mg/L). Semaglutide reduced hsCRP levels by clinical cutoffs and tertiles from baseline to end-of-treatment in all trials versus comparators (estimated treatment ratios [ETRs] versus comparators: 0.70–0.76; p < 0.01) except versus placebo in PIONEER 5 (ETR [95% CI]: 0.83 [0.67–1.03]; p > 0.05). The effect of semaglutide on hsCRP was partially mediated (20.6–61.8%) by change in HbA_1c_ and BW.

**Conclusions:**

Semaglutide reduced hsCRP ratios-to-baseline versus comparators in subjects with type 2 diabetes (not significant with CKD). This effect was partially mediated via reductions in HbA_1c_ and BW and potentially by a direct effect of semaglutide. Semaglutide appears to have an anti-inflammatory effect, which is being further investigated in ongoing trials.

*Trial registrations:* ClinicalTrials.gov identifiers: NCT01885208 (first registered June 2013), NCT02906930 (first registered September 2016), NCT02863328 (first registered August 2016), NCT02827708 (first registered July 2016).

**Supplementary Information:**

The online version contains supplementary material available at 10.1186/s12933-022-01585-7.

## Background

Chronic, low-grade, systemic inflammation is a feature of type 2 diabetes, identifiable by the presence of circulating inflammatory markers [1, 2]. Elevated plasma glucose levels and dyslipidemia have been associated with proinflammatory cytokine production, suggesting a mechanistic link between type 2 diabetes, inflammation, and atherosclerotic cardiovascular (CV) disease [1, 2], and this inflammatory state may contribute to the development and progression of type 2 diabetes [2]. High-sensitivity C-reactive protein (hsCRP) is a sensitive biomarker of systemic inflammation reported to be elevated in patients with type 2 diabetes with other underlying comorbidities [3, 4]. The effects of statins on hsCRP have been studied in the JUPITER (Justification for the Use of Statins in Prevention: an Intervention Trial Evaluating Rosuvastatin) trial, which enrolled patients based on hsCRP levels, and showed that rosuvastatin reduced hsCRP levels and resulted in fewer CV events compared with placebo [5]. This has been confirmed in a recent meta-analysis that showed statins reduce hsCRP levels in patients with CV disease [6]. According to the Centers for Disease Control and Prevention/American Heart Association guidelines, hsCRP < 1 mg/L indicates low CV risk, hsCRP of 1–3 mg/L indicates intermediate CV risk, and hsCRP > 3 mg/L indicates high CV risk [7]. These hsCRP categories are also used to predict CV outcomes [8].

Semaglutide, a human glucagon-like peptide-1 receptor agonist (GLP-1RA) available in weekly subcutaneous (s.c.) and daily oral formulations, was investigated in the Semaglutide Unabated Sustainability in Treatment of Type 2 Diabetes (SUSTAIN) and Peptide Innovation for Early Diabetes Treatment (PIONEER) phase 3 clinical trial programs, against placebo and active comparators [9–13]. The CV outcomes trials, SUSTAIN 6 and PIONEER 6, which were part of these programs, demonstrated CV benefits with s.c. semaglutide and CV non-inferiority with oral semaglutide vs placebo, respectively [14, 15]. Although there have been several studies with GLP-1RAs demonstrating a significant reduction in hsCRP [16, 17], the impact of semaglutide on hsCRP has yet to be fully elucidated.

The aim of this exploratory analysis was to evaluate the effect of semaglutide on hsCRP, and the contribution of glucose control and weight loss to this effect, in subjects at different stages of type 2 diabetes.

## Methods

### Trial designs and patient populations

This analysis included phase 3 trials from the semaglutide (s.c. and oral) clinical development programs that collected hsCRP data, specifically SUSTAIN 3 and PIONEER 1, 2, and 5 [18–21]; hsCRP data collected in SUSTAIN 2, 4, and 5 trials [22–24] were not used due to technical issues during sample collection — the hsCRP analysis assay was found to have a negative bias (an under-recovery of up to –25% at hsCRP concentrations below 5 mg/L), which affected a proportion of the samples.

Study designs for these double-blind, randomized controlled trials have been published previously [18–21]. In brief, subjects with inadequately controlled type 2 diabetes were randomized to receive: once-weekly s.c. semaglutide 1.0 mg or once-weekly GLP-1RA exenatide extended-release (exenatide ER) 2.0 mg (SUSTAIN 3); once-daily oral semaglutide 3, 7, or 14 mg or placebo (PIONEER 1); once-daily oral semaglutide 14 mg or once-daily sodium–glucose cotransporter-2 (SGLT-2) inhibitor empagliflozin 25 mg (PIONEER 2); and once-daily oral semaglutide 14 mg or placebo (PIONEER 5) [18–21]. Treatment durations were 56 weeks (SUSTAIN 3), 52 weeks (PIONEER 2), and 26 weeks (PIONEER 1 and 5). All subjects in PIONEER 5 had chronic kidney disease (CKD, estimated glomerular filtration rate [eGFR] 30–59 mL/min/1.73 m^2^ only). Only approved maintenance doses of semaglutide were included in this analysis.

All trial protocols were approved by local independent ethics committees and institutional review boards at each site and trials were conducted in accordance with the Declaration of Helsinki and International Council for Harmonisation of Technical Requirements for Pharmaceuticals for Human Use. All subjects provided written, informed consent prior to trial start.

hsCRP was measured at weeks 0 and 56 in SUSTAIN 3; weeks 0 and 26 in PIONEER 1; weeks 0, 26, and 52 in PIONEER 2; and weeks 0, 8, and 26 in PIONEER 5. Data were evaluated for subjects receiving trial drug without rescue medication (trial product estimand), to avoid confounding effects from other antihyperglycemic drugs. Data from SUSTAIN 3, PIONEER 1, and PIONEER 2 were analyzed individually for change in hsCRP but were pooled for baseline characteristics; PIONEER 5 data were analyzed separately because they included subjects with CKD (baseline mean eGFR 48 mL/min/1.73 m^2^) who had longer disease duration (mean 14 years) [21].

### Baseline hsCRP categories

Baseline characteristics were analyzed by hsCRP clinical cutoff (< 1.0, ≥ 1.0 to ≤ 3.0, or > 3.0 mg/L) to identify differences or similarities between subgroups, using combined data for SUSTAIN 3, PIONEER 1, and PIONEER 2; for PIONEER 5, they were calculated separately. Comparisons between baseline hsCRP clinical cutoff subgroups were conducted using a chi-squared test for categorical parameters and a Kruskal–Wallis test for continuous parameters.

### Handling of hsCRP values below lower limit of quantification

If hsCRP values were below the lower limit of quantification (LLoQ), a prespecified imputation rule was applied so that hsCRP values were imputed as LLoQ/2. In all three PIONEER trials, LLoQ was 0.1 mg/L and in SUSTAIN 3, it was 1.4 nmol/L (approx. 0.147 mg/L). The proportion of values that were below LLoQ ranged from 0–0.19% at baseline to 0–0.7% at end-of-treatment.

### Analysis of change in hsCRP from baseline

Because type 2 diabetes is a pro-inflammatory disease and patients may therefore have background chronic inflammation, hsCRP data were analyzed as ratios to baseline at end-of-treatment, by baseline clinical cutoff subgroups (< 1.0, ≥ 1.0 to ≤ 3.0, or > 3.0 mg/L) and by hsCRP tertile. Furthermore, to account for potential sex-related differences on the inflammatory response [25], a sensitivity analysis for the effect of treatment (semaglutide or comparator) on ratios-to-baseline by sex (male/female) at baseline was also conducted. Finally, to account for the effect of statins, which are known to reduce hsCRP levels [5, 6], a sensitivity analysis was conducted for the effect of treatment (semaglutide or comparators) on ratios to baseline by statin use (yes/no) at baseline. Absolute mean changes were also assessed by treatment arm in each trial, but not for subgroups because absolute data are more affected by high variability and outliers. Ratios to baseline at end-of-treatment were analyzed by baseline eGFR for PIONEER 5.

Due to right-skewing of the data, hsCRP ratios to baseline at end-of-treatment (Y_eot_/baseline, where Y_eot_ indicates the observed value at the end-of-treatment visit) were log-transformed and analyzed using a mixed model for repeated measurements (MMRM) with treatment arm as categorical fixed effect and baseline value (log-transformed) as covariate, all nested within visit, and an unstructured residual covariance matrix. Absolute change from baseline to end-of-treatment (Y_eot_–baseline) was analyzed by the same model using non-transformed data as a supplementary analysis. From these two models, the treatment ratio (semaglutide arms versus comparators) using log-transformed data and treatment difference (semaglutide arms versus comparators) were estimated. The estimated difference and 95% confidence interval (CI) on the log-scale for each treatment arm were calculated using log-transformed data and were transformed back using the exponential function to generate an average ratio-to-baseline and corresponding 95% CI for each treatment arm on the geometric mean scale. Ratios below one indicated reductions in geometric mean hsCRP. Percentage reductions were calculated using 100% multiplied by (1 – ratio-to-baseline).

In all models, the data for comparator arms were pooled. Observed values from the on-treatment without rescue medication period were included in the MMRM. In an MMRM, the missing values are automatically accounted for using a missing-at-random assumption. A p-value of < 0.05 was considered significant; no adjustment for multiplicity was performed.

### Subgroup analyses of change in hsCRP from baseline to end-of-treatment

The subgroup analyses of hsCRP clinical cutoffs (< 1.0, ≥ 1.0 to ≤ 3.0, or > 3.0 mg/L), hsCRP tertiles, sex (male/female), statin use at baseline (yes/no), and baseline eGFR (< 45 or ≥ 45 mL/min/1.73 m^2^; PIONEER 5 data only) were conducted using MMRMs with treatment arm, subgroup, and treatment arm-by-subgroup interaction as fixed effects and baseline hsCRP value (log-transformed) as covariate, all nested within visit, and an unstructured residual/covariance matrix. The interaction p-value was evaluated at end-of-treatment. The proportion of subjects who moved between risk groups (defined as hsCRP ≤ 3.0 or > 3.0 mg/L) was also assessed.

### Mediation analyses

Mediation analyses examine associations among known, measured variables and outcomes, but do not necessarily identify causality. In this analysis, potential mediators for change in hsCRP, adjusted for possible confounders, were investigated. Mediation analyses included changes in HbA_1c_ and body weight (BW) as mediators, separately and combined, for pooled semaglutide arms (7 mg and 14 mg in PIONEER 1) versus comparators.

The direct effect is the effect of semaglutide versus comparator on change in hsCRP, independent of changes in HbA_1c_ and/or BW. This was estimated at end-of-treatment using an MMRM similar to that described above, with log-transformed values of hsCRP adjusted for baseline hsCRP (log-transformed), change of the mediator and baseline of the mediator and treatment, all nested within visit. The indirect effect is the effect of semaglutide versus comparator on change in hsCRP associated with changes in HbA_1c_ and/or BW. This was estimated at end-of-treatment using an MMRM with change in the mediator adjusted for baseline hsCRP (log-transformed) and the baseline of the mediator and treatment and other relevant confounders, all nested within visits. The total effect was calculated as the sum of the direct and indirect effects using the product method. From these two models, the proportion of the effect mediated by change in HbA_1c_ and BW was estimated at end-of-treatment, respectively, as calculated by the indirect effect divided by the total effect × 100. Similarly, the combined indirect effect from change in HbA_1c_ and BW was calculated using each mediator as an endpoint in separate MMRMs (as described above), including all mediators and log-transformed hsCRP as baseline values and other relevant baseline variables (confounders) as presented in VanderWeelle 2014 [26], and from these models the proportion mediated was estimated.

## Results

### Baseline characteristics

A total of 2471 of 2482 randomized subjects (99.6%) had an hsCRP measurement at baseline and were included in the analyses. Geometric mean (% coefficient of variation) baseline hsCRP values were 2.8 (153) mg/L in SUSTAIN 3, 2.9 (173) mg/L in PIONEER 1, 2.7 (171) mg/L in PIONEER 2, and 3.0 (141) mg/L in PIONEER 5. As expected, subjects in the combined SUSTAIN 3, PIONEER 1, and PIONEER 2 analysis were younger, had a shorter disease duration, higher eGFR and lower systolic blood pressure at baseline, and were more likely to be previous or current smokers than subjects with CKD in PIONEER 5. Mean BMI at baseline was similar across all four included trials (Table 1).

**Table 1:** Baseline characteristics by baseline clinical cutoffs (SUSTAIN 3 and PIONEER 1 and 2; PIONEER 5)

	SUSTAIN 3, PIONEER 1 and 2 (pooled)					﻿PIONEER 5				
	Overall﻿	Low cutoff ﻿(< 1.0 mg/L)	Medium cutoff (≥ 1.0 to ≤ 3.0 mg/L)	High cutoff (>﻿ 3.0 mg/L)	p-value	Overall	Low cutoff (< 1.0 mg/L)	Medium cutoff (≥ 1.0 to ≤ 3.0 mg/L)	High cutoff ﻿(> 3.0 mg/L)	p-value
Full analysis set, N	2158					324﻿				
Randomized with hsCRP measured at baseline, n	2150	394	750	1006		321	45	118	158	
Age, years	56.4 ± 10.5	59.0 ± 9.6	57.6 ± 10.0	54.6 ± 10.9	< 0.0001	70.4 ± 7.9	72.4 ± 6.3	70.9 ± 8.1	69.4 ± 8.0	0.0422
Female sex, n (%)	1028 (47.6)	114 (28.9)	305 (40.7)	605 (60.1)	< 0.0001	168 (51.9)	17 (37.8)	58 (49.2)	91 (57.6)	0.0498
HbA_1c_, %	8.2 ± 0.9	8.0 ± 0.8	8.1 ± 0.9	8.2 ± 0.9	0.0009	8.0 ± 0.7	7.9 ± 0.6	8.0 ± 0.6	8.0 ± 0.8	0.6179
Body weight, kg	92.4 ± 21.5	82.7 ± 17.2	90.0 ± 19.5	98.0 ± 22.6	< 0.0001	90.8 ± 17.6	85.0 ± 13.5	89.9 ± 15.4	93.2 ± 19.7	0.0653
Body mass index, kg/m^2^	32.9 ± 6.5	28.7 ± 4.4	31.7 ± 5.4	35.5 ± 6.8	< 0.0001	32.4 ± 5.4	29.5 ± 3.8	31.9 ± 4.9	33.5 ± 5.8	< 0.0001
Diabetes duration, years	7.1 ± 6.3	8.5 ± 6.5	7.7 ± 6.8	6.1 ± 5.6	< 0.0001	14.0 ± 8.0	13.6 ± 6.4	15.0 ± 8.4	13.4 ± 8.1	0.1453
Previous/current smoker, n (%)	933 (43.2)	184 (46.7)	310 (41.3)	437 (43.4)	0.2181	113 (34.9)	21 (46.7)	36 (30.5)	55 (34.8)	0.1537
eGFR (CKD-EPI), mL/min/1.73 m^2^	96.7 ± 15.3	95.1 ± 13.2	95.1 ± 15.0	98.4 ± 16.1	< 0.0001	47.6 ± 9.7	50.6 ± 8.5	47.2 ± 9.8	47.1 ± 9.9	0.0485
eGFR, n (%)										0.0612
≥ 45 mL/min/1.73 m^2^	N/A*	N/A*	N/A*	N/A*		202 (62.3)	34 (75.6)	66 (55.9)	101 (63.9)	
< 45 mL/min/1.73 m^2^	N/A*	N/A*	N/A*	N/A*		122 (37.7)	11 (24.4)	52 (44.1)	57 (36.1)	
Lipids, mmol/L										
Total cholesterol	4.8 ± 1.1	4.6 ± 1.0	4.8 ± 1.1	4.9 ± 1.1	< 0.0001	4.6 ± 1.3	4.2 ± 1.0	4.3 ± 1.1	4.9 ± 1.4	0.0002
HDL cholesterol	1.2 ± 0.3	1.2 ± 0.3	1.2 ± 0.3	1.2 ± 0.3	0.0019	1.1 ± 0.3	1.2 ± 0.4	1.1 ± 0.3	1.1 ± 0.2	0.7598
LDL cholesterol	2.7 ± 0.9	2.5 ± 0.9	2.7 ± 0.9	2.8 ± 0.9	< 0.0001	2.5 ± 1.0	2.3 ± 0.9	2.3 ± 0.9	2.7 ± 1.1	0.0008
Triglycerides	2.1 ± 1.4	1.8 ± 1.1	2.1 ± 1.4	2.2 ± 1.4	< 0.0001	2.3 ± 1.4	1.9 ± 1.0	2.1 ± 1.1	2.6 ± 1.6	0.0009
Diastolic BP, mmHg	80.1 ± 9.0	79.0 ± 9.3	80.2 ± 8.6	80.4 ± 9.1	0.0215	77.6 ± 9.1	76.0 ± 9.0	77.8 ± 8.7	77.8 ± 9.5	0.5244
Systolic BP, mmHg	132.2 ± 14.7	131.8 ± 14.9	132.8 ± 14.3	131.8 ± 14.8	0.2370	137.5 ± 15.1	138.4 ± 17.8	137.6 ± 13.4	137.4 ± 15.7	0.9382
Statin use at baseline, yes, n (%)	891 (41.3)	194 (49.2)	316 (42.1)	377 (37.5)	0.0003	234 (72.2)	39 (86.7)	87 (73.7)	107 (67.7)	0.0400

^*^In SUSTAIN 3, PIONEER 1, and PIONEER 2, subjects with a baseline eGFR of < 60 mL/min/1.73 m^2^ were excluded. Data are means (standard deviation) for continuous parameters and n (%) for categorical parameters. Percentages are based on total number of subjects. P-values for tests of differences between subgroups regardless of treatment group have been taken from a chi-squared test for categorical parameters and from a Kruskal–Wallis test for continuous parameters. *BP* blood pressure; *CKD-EPI* Chronic Kidney Disease–Epidemiology Collaboration; *eGFR* estimated glomerular filtration rate; *HbA*_*1c*_ glycated hemoglobin; *HDL* high-density lipoprotein; *hsCRP* high-sensitivity C-reactive protein; *LDL* low-density lipoprotein; *n* number of subjects with available hsCRP data; *N* full analysis set; *N/A* not applicable

In SUSTAIN 3, PIONEER 1, PIONEER 2, and PIONEER 5, there were more subjects in the high clinical cutoff group (> 3.0 mg/L) than in each of the lower cutoff (< 1.0 and ≥ 1.0 to ≤ 3.0 mg/L) groups. Subjects in this group in the SUSTAIN 3, PIONEER 1, and PIONEER 2 populations were younger, with a higher HbA_1c_, BW, BMI, and eGFR, and a shorter diabetes duration versus those in lower clinical cutoff groups. In PIONEER 5, subjects in the high clinical cutoff were younger, had a higher BMI and a lower eGFR versus those in the lower clinical cutoffs.

### Estimated treatment ratios and ratio-to-baseline at end-of-treatment by trial

**Fig. 1:**
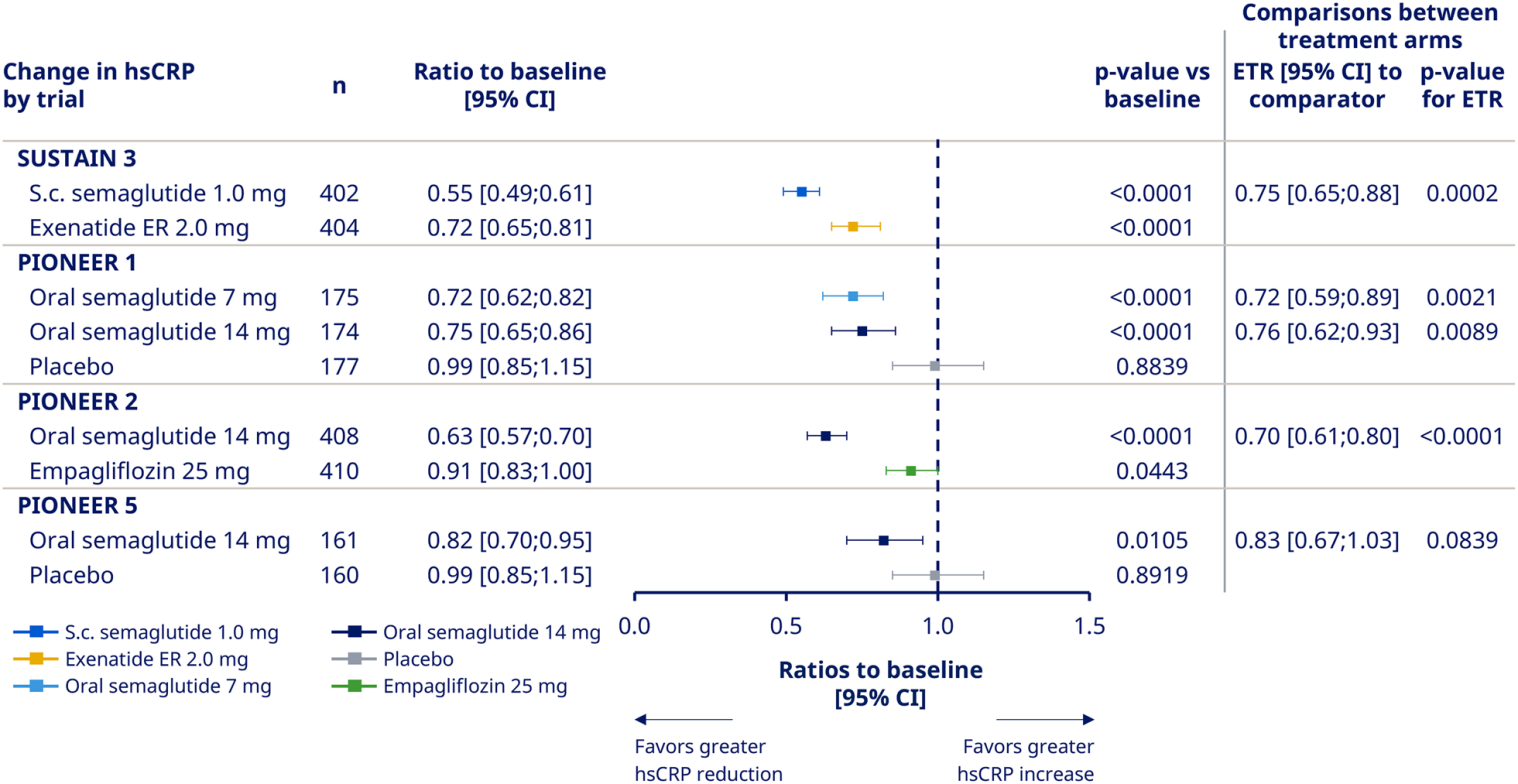
Ratios to baseline at end-of-treatment for hsCRP with semaglutide and comparators by trial. ‘On-treatment without rescue medication’ data from the full analysis set. Ratios to baseline were analyzed using a mixed model for repeated measurements with treatment as categorical fixed effect and baseline hsCRP value (log-transformed) as covariate, all nested within visit, and an unstructured residual covariance matrix on log-transformed values. *CI* confidence interval; *exenatide ER* exenatide extended-release; *ETR* estimated treatment ratio; *hsCRP* high-sensitivity C-reactive protein; *N* number of subjects with available hsCRP data; *s.c.* subcutaneous

In SUSTAIN 3, PIONEER 1, and PIONEER 2, there was a significant reduction in hsCRP from baseline to end-of-treatment with semaglutide versus comparators. In SUSTAIN 3, the estimated treatment ratio (ETR) at week 56 for semaglutide 1.0 mg versus exenatide ER 2.0 mg was reduced by 25% (0.75 [95% CI, 0.65–0.88]; p < 0.001; Fig. 1). In PIONEER 1, the ETRs at week 26 for oral semaglutide 7 mg and 14 mg versus placebo were reduced by 28% and 24% (0.72 [95% CI, 0.59–0.89]; p < 0.01 and 0.76 [95% CI, 0.62–0.93]; p < 0.01, respectively; Fig. 1). In PIONEER 2, the ETR for semaglutide 14 mg versus empagliflozin 25 mg at week 52 was reduced by 30% (0.70 [95% CI, 0.61–0.80]; p < 0.0001; Fig. 1). In PIONEER 5, there was a nonsignificant trend of reduction, as the ETR at week 26 for oral semaglutide versus placebo was 0.83 [95% CI, 0.67–1.03]; p = 0.08; Fig. 1.

Reductions of hsCRP from baseline were significant in all trials with semaglutide (ratio-to-baseline at end-of-treatment 0.55 to 0.82, p ≤ 0.01 for all), and with the two active comparators, exenatide ER and empagliflozin (ratio-to-baseline at end-of-treatment 0.72 and 0.91, p < 0.0001 and p = 0.0443, respectively; Fig. 1). hsCRP was not changed from baseline in the placebo arms of the trials with a ratio-to-baseline of 0.99 in both trials (Fig. 1).

### Estimated treatment ratios and ratio-to-baseline by trial and subgroups

#### Clinical cutoffs of hsCRP at baseline

**Fig. 2:**
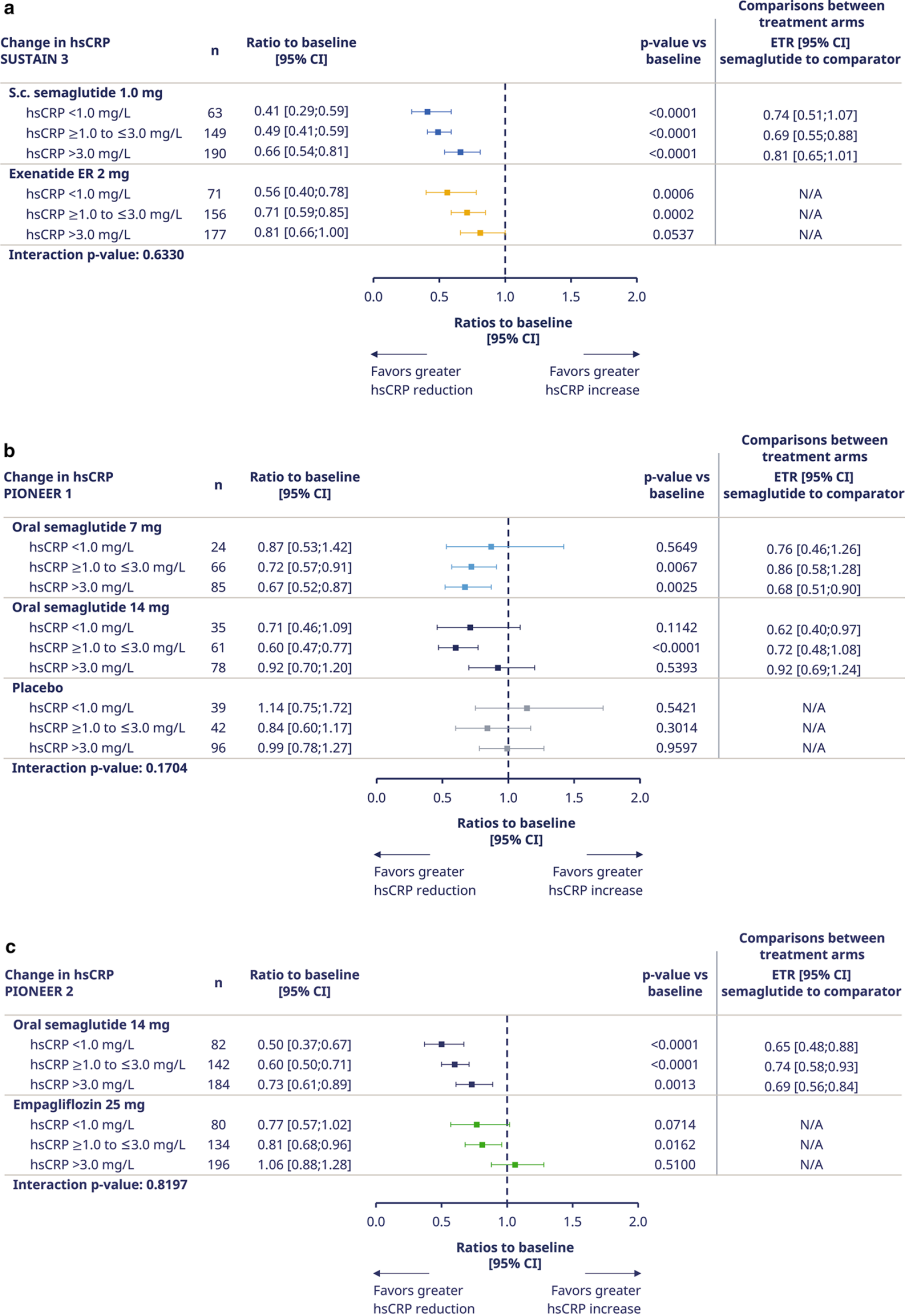

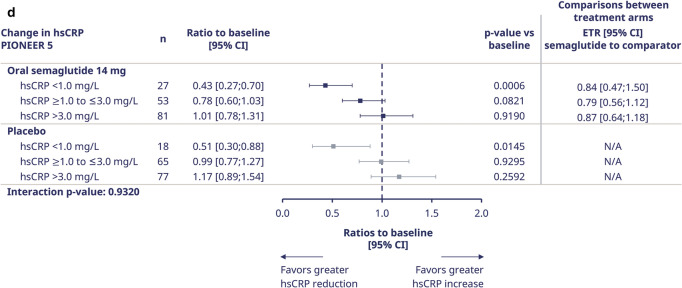
Ratio to baseline at end-of-treatment for hsCRP by trial according to clinical cutoffs. Panel (a) shows SUSTAIN 3 data, panel (b) PIONEER 1 data, panel (c) PIONEER 2 data, and panel (d) PIONEER 5 data. ‘On-treatment without rescue medication’ data from the full analysis set. Ratios to baseline were analyzed using a mixed model for repeated measurements with treatment by hsCRP groups as categorical fixed effects and baseline hsCRP value (log-transformed) as covariate, all nested within visit, and an unstructured residual covariance matrix on log-transformed values. Clinical cut-offs used in this analysis were < 1.0, ≥ 1.0 to ≤ 3.0, and > 3.0 mg/L. *CI* confidence interval; *ETR* estimated treatment ratio; *exenatide ER* exenatide extended-release; *hsCRP* high-sensitivity C-reactive protein; *N * number of subjects with available hsCRP data; *s.c.* subcutaneous

In all trials, the ETRs between semaglutide versus comparators were not affected by hsCRP cutoff group at baseline, as indicated by the nonsignificant interaction p-values for each trial (Fig. 2).

#### hsCRP tertiles at baseline

In all trials, the ETRs for semaglutide versus comparators were not affected by hsCRP tertile at baseline, as indicated by the interaction p-values for each trial, which were all nonsignificant, except for PIONEER 1 (p_interaction_: 0.0074; Additional file 1: Fig. S1). The trend was for ratios-to-baseline at end-of-treatment to be lowest in the lowest tertiles for semaglutide and for the active comparators, i.e. the largest hsCRP reductions from baseline were observed in subjects with the lowest baseline hsCRP levels; the ratio-to-baseline increased progressively in the middle and high hsCRP tertile groups (Additional file 1: Fig. S1).

#### Stratification by sex

Sex (female or male) did not affect the ETRs for semaglutide versus comparators, as indicated by the interaction p-values in any of the trials (all p_interaction_ > 0.05) (Additional file 1: Fig. S2).

#### Statin use at baseline

Statin use at baseline (yes or no) did not affect the ETRs for semaglutide versus comparators, as indicated by the nonsignificant interaction p-values for each trial (Additional file 1: Fig. S3). No clear pattern in ratio-to-baseline was observed according to statin use at baseline for semaglutide and all comparators.

#### eGFR (< 45 versus ≥ 45 mL/min/1.73 m^2^) at baseline

eGFR group at baseline (< 45 or ≥ 45 mL/min/1.73 m^2^) did not affect the ETRs for semaglutide versus placebo in PIONEER 5. The ETR for semaglutide versus placebo for eGFR < 45 mL/min/1.73 m^2^ was 0.91 [95% CI, 0.64–1.30] and for eGFR ≥ 45 mL/min/1.73 m^2^ was 0.78 [95% CI, 0.59–1.02]. The p_interaction_ between treatment and eGFR subgroup was 0.48 (data not shown).

### hsCRP cutoff at end-of-treatment versus baseline

In most study arms, a greater proportion of subjects moved to the ≤ 3.0 mg/L hsCRP category from the > 3.0 mg/L hsCRP category than moved in the opposite direction (this was not observed with placebo in PIONEER 5). The percentage of subjects moving from baseline hsCRP ≤ 3.0 mg/L to hsCRP > 3.0 mg/L at end-of-treatment was greater with comparators (exenatide ER, empagliflozin, and placebo) compared with semaglutide (except with oral semaglutide 7 mg versus placebo in PIONEER 1), and the percentage of subjects moving from baseline hsCRP > 3.0 mg/L to hsCRP ≤ 3.0 mg/L at end-of-treatment was greater with semaglutide compared with comparators (Additional file 1: Table S1).

### Absolute hsCRP change from baseline

hsCRP was significantly reduced with oral semaglutide 7 mg versus placebo in PIONEER 1 (estimated treatment difference: −1.39 mg/L [95% CI, −2.56 to −0.22]; Additional file 1: Fig. S4). In the other trials, there was a trend for a negative absolute hsCRP change from baseline with oral semaglutide 14 mg versus placebo (PIONEER 5) and with semaglutide (s.c. 1.0 mg or oral 14 mg doses) versus active comparators (SUSTAIN 3 and PIONEER 2, respectively), although these differences did not reach statistical significance (p > 0.05; Additional file 1: Fig. S4).

### Mediation analyses

**Fig. 3:**
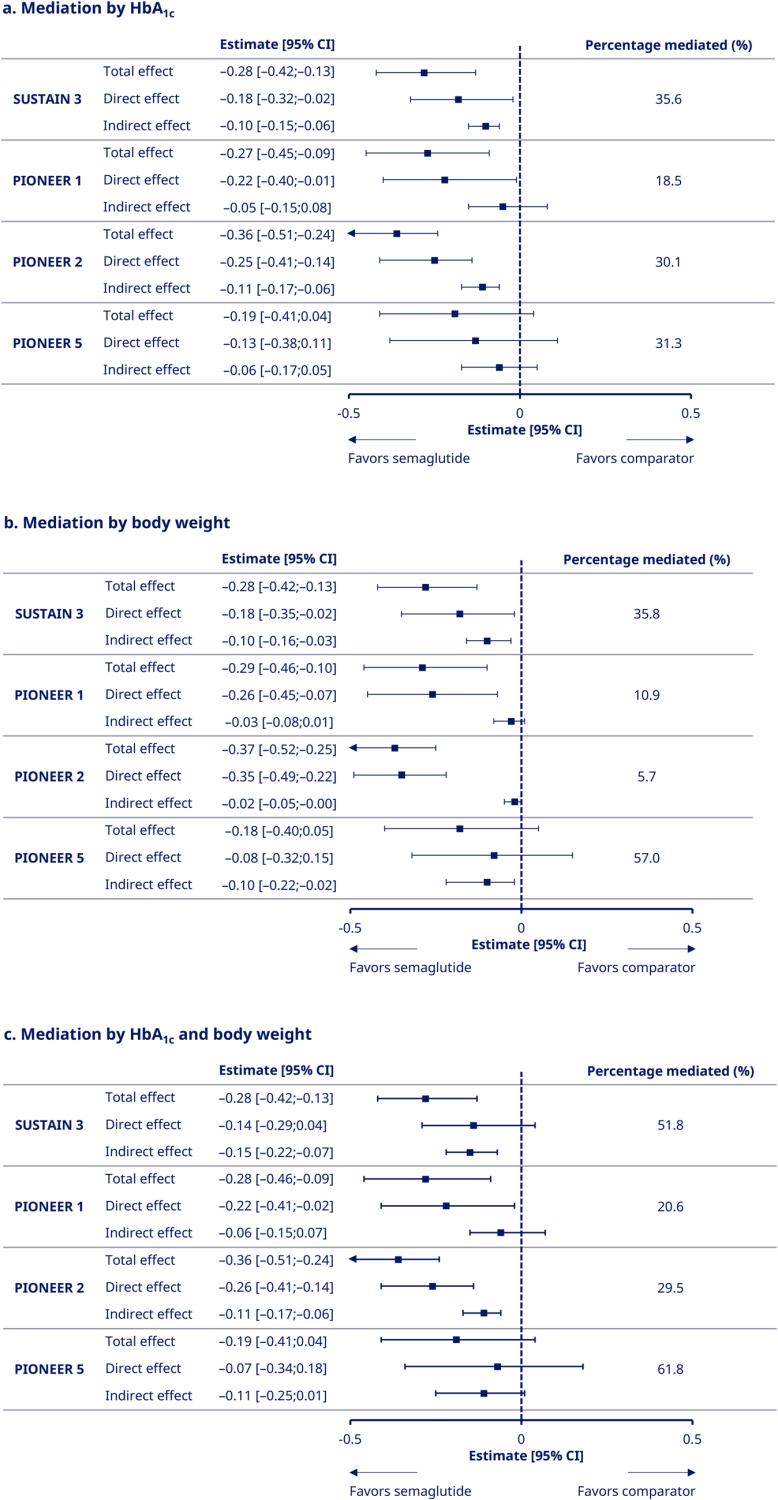
Mediation of hsCRP by change in HbA_1c_ and/or body weight. Panel (a) shows mediation of hsCRP by HbA_1c_, panel (b) by body weight, and panel (c) by HbA_1c_ and body weight combined, all according to trial and with pooled semaglutide arms versus comparators. The indirect effect is the effect of semaglutide versus comparator on change in hsCRP that was caused by its effect through change on HbA_1c_ and/or BW. The direct effect is the effect of semaglutide versus comparator on change in hsCRP independent of change in HbA_1c_ and/or BW. The total effect was calculated by adding the indirect effect to the direct effect. The proportion mediated was calculated by dividing the indirect effect by the total effect × 100. Data from the ‘on-treatment without rescue medication’ period. Mediation was analyzed with mixed models for repeated measurements, adjusted for relevant mediators at baseline: HbA_1c_ (panel a), BW (panel b), BW and HbA_1c_ (panel c); and furthermore adjusted for additional covariates at baseline: log(hsCRP), age, sex, eGFR (Chronic Kidney Disease–Epidemiology Collaboration 2009), body mass index, statins (yes/no), insulin treatment (PIONEER 5 analyses only), metformin treatment (SUSTAIN 3 and PIONEER 5 analyses only), and HbA_1c_ (panel b only). Mediator data were only taken from the visits where hsCRP was assessed for estimation of the direct effect. *BW* body weight; *CI* confidence interval; *eGFR* estimated glomerular filtration rate; *HbA*_*1c*_ glycated hemoglobin; *hsCRP* high-sensitivity C-reactive protein

Some of the effect of semaglutide (s.c. 1.0 mg, oral 7 mg, and oral 14 mg) on hsCRP was shown to be mediated by change in HbA_1c_ (18.5–35.6%; Fig. 3a), with change in BW playing a lesser role in PIONEER 1 (10.9%) and PIONEER 2 (5.7%) but a similar or greater role in SUSTAIN 3 (35.8%) and PIONEER 5 (57.0%; Fig. 3b). When the two mediation parameters were considered together, some but not all of the effect of semaglutide on hsCRP was mediated by a change in HbA_1c_ and BW (combined percentage mediated: 20.6–61.8%; Fig. 3c). The sum of the proportions mediated individually by change in BW and change in HbA_1c_ was greater than the combined mediated proportion, which is likely to be caused by associations between HbA_1c_ and BW.

## Discussion

This exploratory analysis suggests that s.c. and oral semaglutide significantly reduced hsCRP levels versus comparators in subjects with type 2 diabetes. The comparators were placebo in PIONEER 1 and PIONEER 5, and active comparators in SUSTAIN 3 (exenatide ER) and PIONEER 2 (empagliflozin). The effect of semaglutide on hsCRP was partially mediated via reductions in HbA_1c_ and BW, which may therefore be involved in a potential anti-inflammatory effect of semaglutide. However, there may also be a direct effect of semaglutide on hsCRP, supporting potential direct anti-inflammatory action.

When stratified by hsCRP clinical cutoffs (< 1.0 mg/L, ≥ 1.0 to ≤ 3.0 mg/L, > 3.0 mg/L), subjects in the highest clinical cutoff were younger, with higher HbA_1c_, BW, and BMI (and higher eGFR in the pooled SUSTAIN 3, PIONEER 1 and 2 analysis only) at baseline. Treatment ratio reductions were generally similar across these categories, although some ETRs were not significant, possibly due to a lack of statistical power resulting from small sample size. Treatment ratios were also generally similar when subjects were stratified by tertile, sex, baseline statin use, or eGFR (data from PIONEER 5 only). Furthermore, more subjects treated with semaglutide moved from a higher to a lower hsCRP category compared with those treated with comparators, possibly indicating reduced inflammation. Finally, reductions in hsCRP appeared to be partially associated with change in HbA_1c_ and also in BW (although to a lesser extent in PIONEER 1 and 2 than in the other trials); however, there are some supporting data for a direct effect of semaglutide.

There are several analyses of the effects of GLP-1RAs on hsCRP. Decreases in hsCRP have also been observed with other classes of antihyperglycemic drug (metformin, dipeptidyl peptidase-4 inhibitors, and SGLT-2 inhibitors), but with less consistency than with GLP-1RAs [27]. The beneficial effect of GLP-1RAs on hsCRP has been shown in a meta-analysis of small trials with liraglutide and exenatide [28]. In addition, analysis of a 52-week phase 2 trial investigated once-daily ≤ 0.4 mg doses of s.c. semaglutide (N = 957) and the STEP phase 3 trials investigated s.c. semaglutide 2.4 mg dose in subjects with overweight and obesity with or without type 2 diabetes (total N = 4684); these trials identified consistent reductions in hsCRP with once-daily semaglutide ≤ 0.4 mg and once-weekly s.c. semaglutide 2.4 mg [28–32]. An analysis of subjects with type 2 diabetes and obesity in the LYDIA trial found there was no significant difference in the effects of liraglutide on hsCRP compared with the dipeptidyl peptidase-4 inhibitor sitagliptin [33]. However, a recent meta-analysis, which included data from 40 randomized controlled trials in subjects with type 2 diabetes, found significant improvements with GLP-1RAs compared with standard diabetes therapies (including sulfonylureas, insulins, dipeptidyl peptidase-4 inhibitors, thiazolidinediones) and placebo in several clinical biomarkers, including CRP, tumor necrosis factor-alpha, and adiponectin (markers of inflammation) and malondialdehyde (a marker of oxidative stress) [34]. Our analyses not only confirm previous findings of hsCRP reductions with a GLP-1RA, but also expand knowledge on these reductions, which can occur regardless of baseline hsCRP, sex, and statin use. Reductions in hsCRP are highly clinically relevant: elevated hsCRP has been found to be predictive of major adverse CV events and death in individuals with or without type 2 diabetes and/or previous CV events [35]. The value of hsCRP as a CV risk factor appears similar to that of conventional CV risk factors – hsCRP is not a causal factor for CV disease and serves as a biomarker only [36].

As inflammation is an important process in CV disease [1, 2] and GLP-1RAs are hypothesized to lower CV risk via attenuation of atherosclerosis [37, 38], the hsCRP reduction observed with the GLP-1RA semaglutide suggests that it might exert anti-inflammatory effects. Some possible mechanisms for this action include GLP-1 receptor expression in the intestine, which would lead to an improved gut barrier function [39, 40], or reprogramming of macrophages [41]. However, the actual mechanism remains unknown.

Our analyses contained data from PIONEER 5, which included subjects with CKD. However, as hsCRP and other inflammatory markers are typically higher in patients with CKD [42], the data from PIONEER 5 were analyzed separately to those of the other trials. Mean baseline hsCRP in PIONEER 5 was similar to that in other trials, and hsCRP ratio to baseline was reduced at end-of-treatment. Furthermore, baseline eGFR categories were shown to have no effect on the hsCRP ratio to baseline at end-of-treatment. This is a clinically relevant result, because reductions in hsCRP have been linked to improved kidney outcomes [43]. In general, the pathological milieu is altered in patients with kidney impairment and may lead to differences in responses to drugs [44], although the renal safety of semaglutide presented in the PIONEER 5 primary publication was consistent with the suggested kidney-protective effects of GLP-1RAs overall in this population [21].

In the mediation analyses, which examined change in HbA_1c_ and BW (both separately and combined) as potential mediators for the effect of semaglutide on hsCRP, the effect on hsCRP was partially mediated via the effect of semaglutide on HbA_1c_ and BW, although HbA_1c_ seemed to contribute slightly more than BW. Our findings are consistent with those from a longitudinal observational study in patients without diabetes, which found that there was a positive association between HbA_1c_ and hsCRP levels [45]. Furthermore, as it is well-known that weight loss has an anti-inflammatory effect [30–32], partial mediation by BW is unsurprising.

Strengths of the current analysis include its large sample size of patient-level data from several randomized clinical trials of semaglutide. Another strength is that the same central laboratory analyzed hsCRP and other laboratory results.

Limitations of this analysis include its exploratory nature, lack of hard endpoints for evaluation, low hsCRP testing frequency, lack of data on other markers of inflammation, and the relatively short follow-up time (26–56 weeks). The analyses were performed using patient-level data for each individual trial as data could not be “pooled” due to differences in trial duration, although pooling was performed for baseline characteristics, with the exception of PIONEER 5 owing to its inclusion of a kidney-impaired population. In addition, hsCRP data are subject to a high degree of variability and outliers may dominate the absolute change in hsCRP data, based on the assumption of a normal distribution. This may explain why differences in the relative change in hsCRP were significant and some absolute changes were not; however, for lower levels of hsCRP at baseline, ratios to baseline may be overly sensitive to small changes. Another limitation is that the mediation analysis did not use all mediator values obtained during the trial that may have a direct relationship with hsCRP levels, as may be done in more complex longitudinal mediation analyses. Finally, these trials were not powered for mediation analyses specifically, and hence there was high variability in the statistics used.

## Conclusions

Semaglutide appeared to reduce hsCRP ratios-to-baseline to a greater extent than comparators — another GLP-1RA (exenatide ER), an SGLT-2 inhibitor (empagliflozin), and placebo (not significant versus placebo in PIONEER 5) — in subjects with type 2 diabetes, and this was partially mediated via its effect on HbA_1c_ and BW. The mediation analysis suggested that there may also be a direct semaglutide effect. Ongoing semaglutide CV and kidney outcome trials, such as SOUL (NCT03914326) investigating subjects with type 2 diabetes and CV disease, SELECT (NCT03574597) investigating subjects with obesity and CV disease, and FLOW (NCT03819153) investigating subjects with type 2 diabetes and renal impairment, may help further elucidate on the semaglutide impact on hsCRP and any possible association between hsCRP reduction and reductions in CV and kidney adverse outcomes. Furthermore, a dedicated CV mode of action trial with semaglutide in subjects with type 2 diabetes and CV disease is also ongoing, which includes inflammation in atherosclerosis as the primary endpoint (NCT04032197).

## Supplementary Information

**Additional file 1: Additional information on SUSTAIN and PIONEER trials. Figure S1.** Ratios to baseline at end-of-treatment for hsCRP with semaglutide and comparators by trial according to baseline tertiles. **Figure S2. **Ratios to baseline at end-of-treatment for hsCRP by trial according to sex (female/male). **Figure S3.** Ratios to baseline at end-of-treatment for hsCRP by trial according to statin use (yes/no). **Figure S4.** Absolute change in hsCRP from baseline to end-of-treatment by trial (semaglutide vs comparator). **Table S1.** Percentage of subjects who moved between hsCRP risk groups according to hsCRP category by trial – change between baseline and end of treatment

## Data Availability

The datasets used and/or analyzed during the current study are available from the corresponding author on reasonable request.

## Abbreviations

BW: Body weight
CI: Confidence interval
CKD: Chronic kidney disease
CV: Cardiovascular
eGFR: Estimated glomerular filtration rate
Exenatide ER: Exenatide extended-release
ETR: Estimated treatment ratio
GLP-1RA: Glucagon-like peptide-1 receptor agonist
HbA_1c_: Glycated hemoglobin
hsCRP: High-sensitivity C-reactive protein
JUPITER: Justification for the Use of Statins in Prevention: an Intervention Trial Evaluating Rosuvastatin
LLoQ: Lower limit of quantification
MMRM: Mixed model for repeated measurements
PIONEER: Peptide Innovation for Early Diabetes Treatment
s.c.: Subcutaneous
SGLT-2: Sodium–glucose cotransporter-2
SUSTAIN: Semaglutide Unabated Sustainability in Treatment of Type 2 Diabetes
Y_eot_: Observed value at the end-of-treatment visit
