# Supplementary material for: Impact of semaglutide on high-sensitivity C-reactive protein: exploratory patient-level analyses of SUSTAIN and PIONEER randomized clinical trials
Source: Cardiovasc Diabetol. 2022 Sep 2;21:172. doi: 10.1186/s12933-022-01585-7 (PMC9440529; doi:10.1186/s12933-022-01585-7)
Supplement: Supplementary file 1 — Additional file 1: Additional information on SUSTAIN and PIONEER trials. Figure S1. Ratios to baseline at end-of-treatment for hsCRP with semaglutide and comparators by trial according to baseline tertiles. Figure S2. Ratios to baseline at end-of-treatment for hsCRP by trial according to sex (female/male). Figure S3. Ratios to baseline at end-of-treatment for hsCRP by trial according to statin use (yes/no). Figure S4. Absolute change in hsCRP from baseline to end-of-treatment by trial (semaglutide vs comparator). Table S1. Percentage of subjects who moved between hsCRP risk groups according to hsCRP category by trial – change between baseline and end of treatment [file 12933_2022_1585_MOESM1_ESM.docx]

**ADDITIONAL MATERIALS**

**Additional information on SUSTAIN and PIONEER trials**

Across the SUSTAIN trials (SUSTAIN 1–5 and 7–10), subcutaneous semaglutide reduced glycated hemoglobin (HbA_1c_; –1.2 to –1.8 %-points) and body weight (BW; –3.5 to –6.5 kg) versus placebo (<–0.1 to –0.1 %-points and –0.9 to –1.4 kg, respectively) and active comparators (–0.5 to –1.4 %-points and +1.2 to –4.2 kg, respectively) from baseline to end-of-treatment [1-4].

Across the multinational PIONEER trials (PIONEER 1–5, 7 and 8), the approved maintenance doses of oral semaglutide, 7 and 14 mg, reduced HbA_1c_ (–0.8 to –1.4 %-points) and BW (–2.0 to –4.3 kg) versus placebo (–0.2 to –0.3 %-points and +0.5 to –1.4 kg, respectively) and active comparators (–0.7 to –0.9 %-points and –0.7 to –3.6 kg, respectively) from baseline to end-of-treatment [5].

**References**

1. Aroda VR, Ahmann A, Cariou B, Chow F, Davies MJ, Jódar E, et al. Comparative efficacy, safety, and cardiovascular outcomes with once-weekly subcutaneous semaglutide in the treatment of type 2 diabetes: Insights from the SUSTAIN 1-7 trials. Diabetes Metab. 2019;45:409-418.

2. Lingvay I, Catarig AM, Frias JP, Kumar H, Lausvig NL, le Roux CW, et al. Efficacy and safety of once-weekly semaglutide versus daily canagliflozin as add-on to metformin in patients with type 2 diabetes (SUSTAIN 8): a double-blind, phase 3b, randomised controlled trial. Lancet Diabetes Endocrinol. 2019;7:834-844.

3. Zinman B, Bhosekar V, Busch R, Holst I, Ludvik B, Thielke D, et al. Semaglutide once weekly as add-on to SGLT-2 inhibitor therapy in type 2 diabetes (SUSTAIN 9): a randomised, placebo-controlled trial. Lancet Diabetes Endocrinol. 2019;7:356-367.

4. Capehorn MS, Catarig AM, Furberg JK, Janez A, Price HC, Tadayon S, et al. Efficacy and safety of once-weekly semaglutide 1.0mg vs once-daily liraglutide 1.2mg as add-on to 1-3 oral antidiabetic drugs in subjects with type 2 diabetes (SUSTAIN 10). Diabetes Metab. 2020;46:100-109.

5. Thethi TK, Pratley R, Meier JJ. Efficacy, safety and cardiovascular outcomes of once-daily oral semaglutide in patients with type 2 diabetes: The PIONEER programme. Diabetes Obes Metab. 2020;22:1263-1277.

**Figure S1.** Ratios to baseline at end-of-treatment for hsCRP with semaglutide and comparators by trial according to baseline tertiles


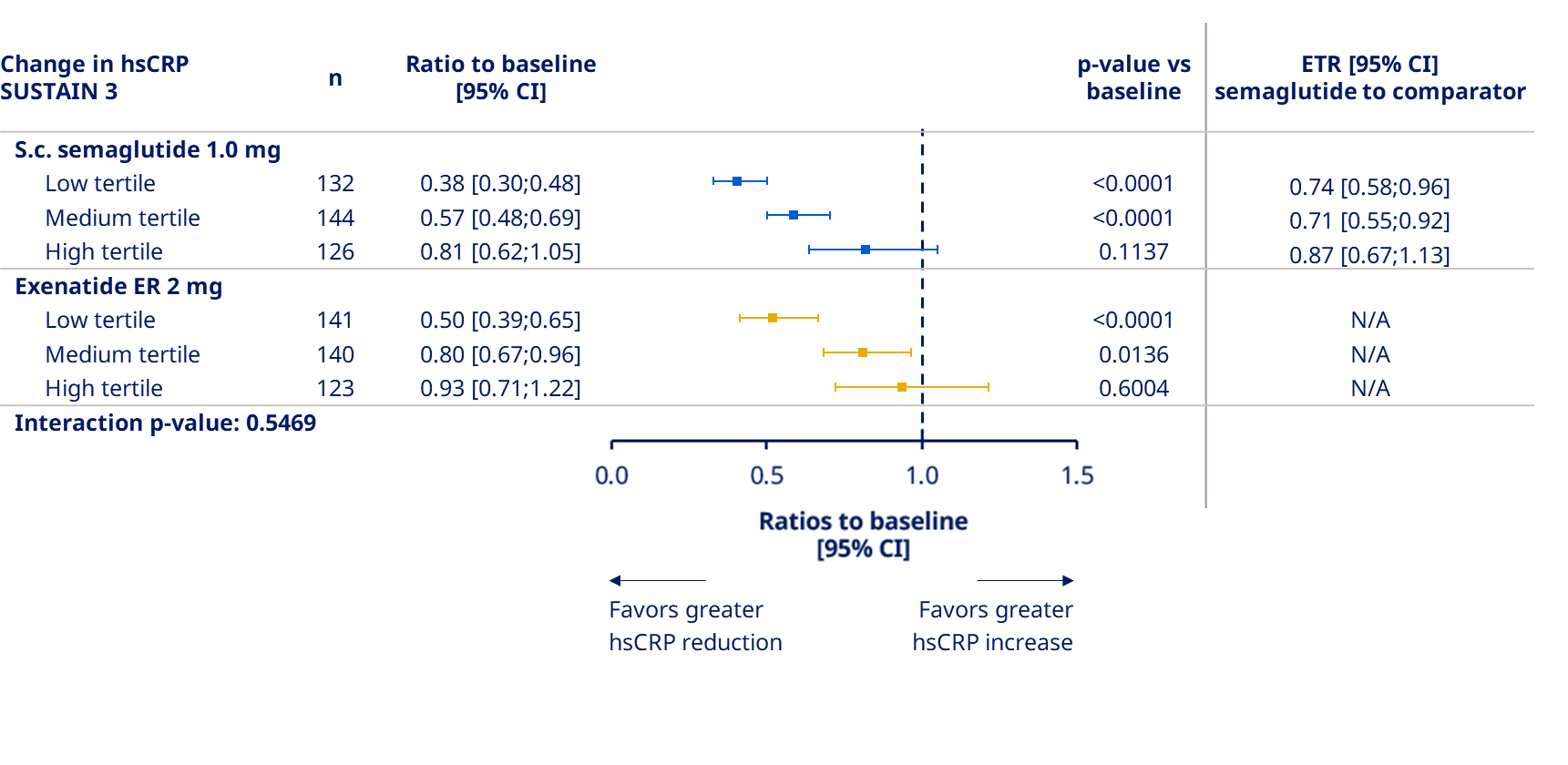


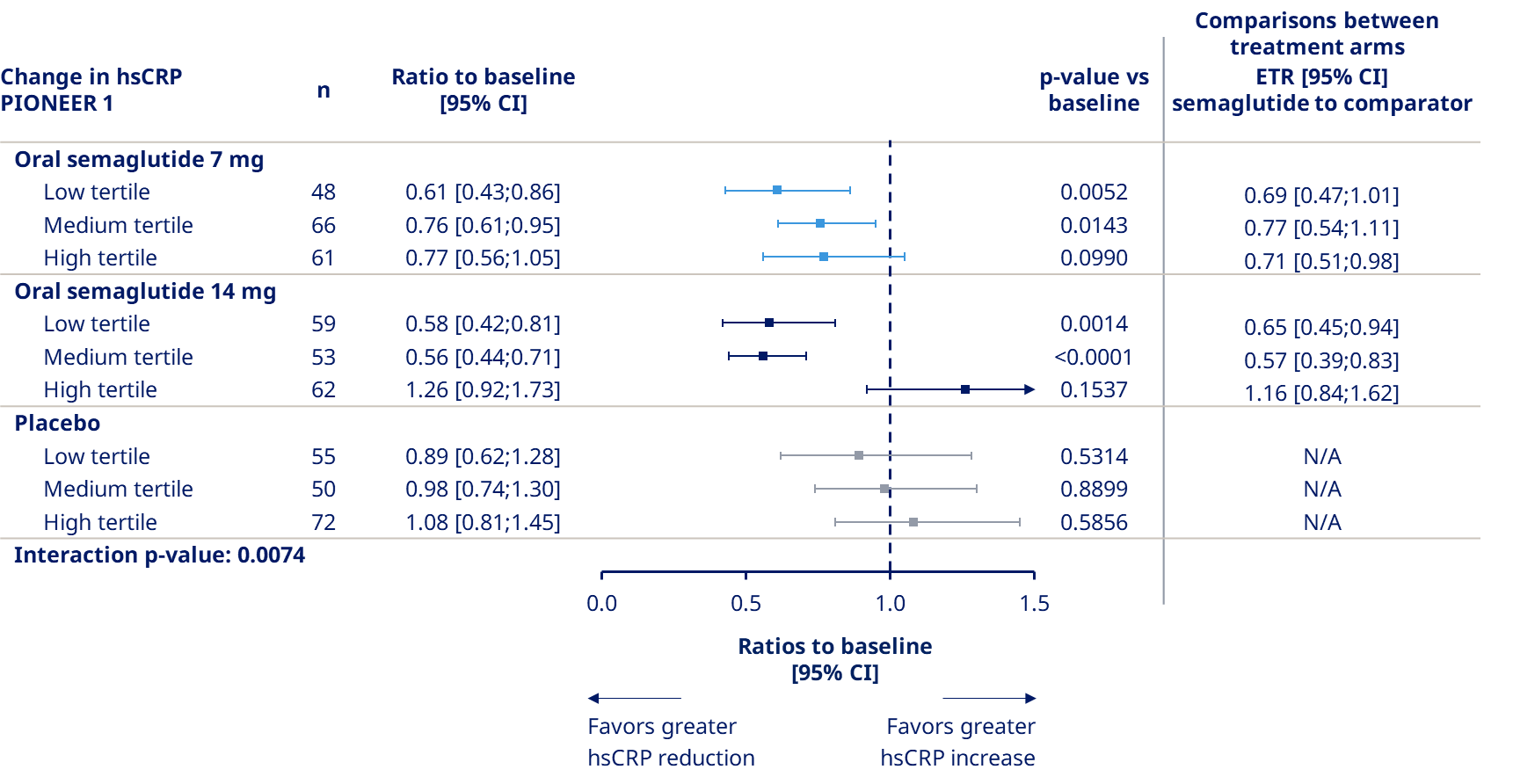


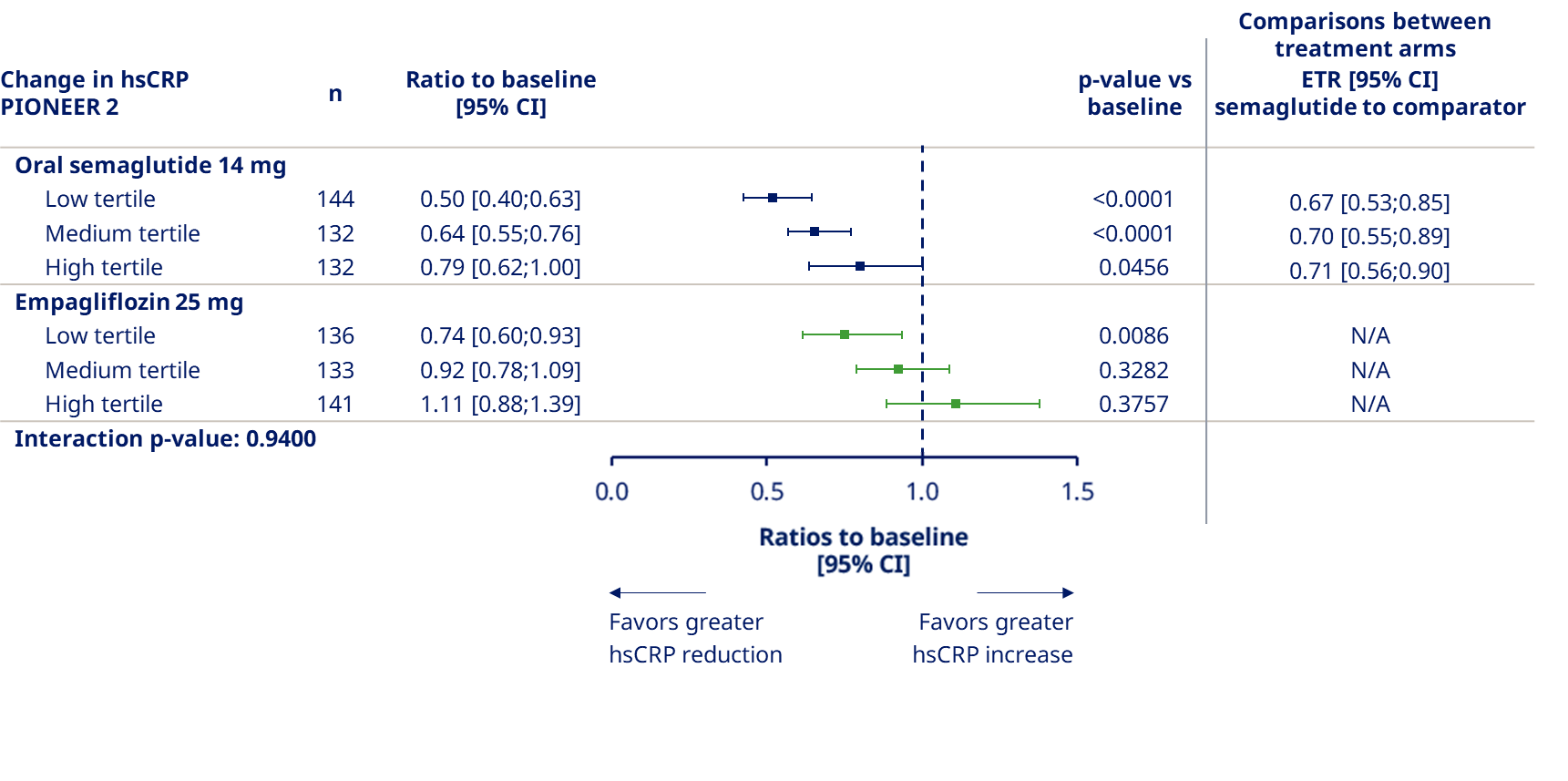


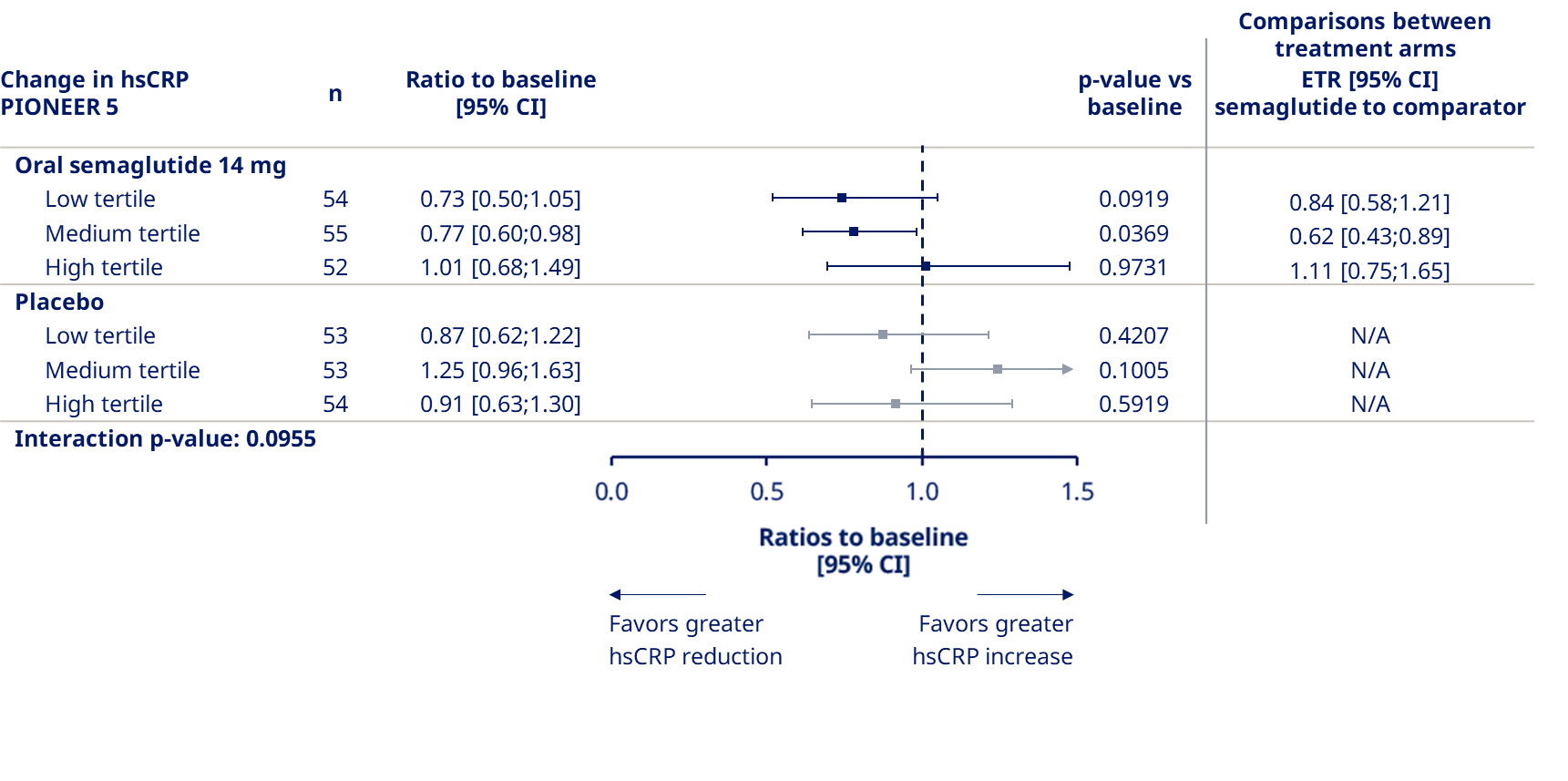


‘On-treatment without rescue medication’ data from the full analysis set. Ratios to baseline were analyzed using a mixed model for repeated measurements with treatment by hsCRP tertiles as categorical fixed effect and baseline value (log-transformed) as covariate, all nested within visit, and an unstructured residual covariance matrix on log-transformed values. To calculate the tertiles, data of subjects from SUSTAIN 3, PIONEER 1 and 2 were pooled; the resulting tertiles that were used in the individual analyses for SUSTAIN 3 and PIONEER 1, 2 were: low tertile hsCRP ≤1.7 mg/L, medium tertile hsCRP 1.7 to ≤4.6 mg/L, high tertile hsCRP >4.6 mg/L. Tertiles for PIONEER 5 were calculated separately, and were: low tertile hsCRP ≤2.0 mg/L, medium tertile hsCRP >2.0 to ≤4.21 mg/L, high tertile hsCRP >4.21 mg/L. *CI* confidence interval; *ETR* estimated treatment ratio; *exenatide ER* exenatide extended release; *hsCRP* high-sensitivity C-reactive protein; *n* number of subjects with available hsCRP data; *N/A* not applicable; *s.c.* subcutaneous

**Figure S2.** Ratios to baseline at end-of-treatment for hsCRP by trial according to sex (female/male)

**
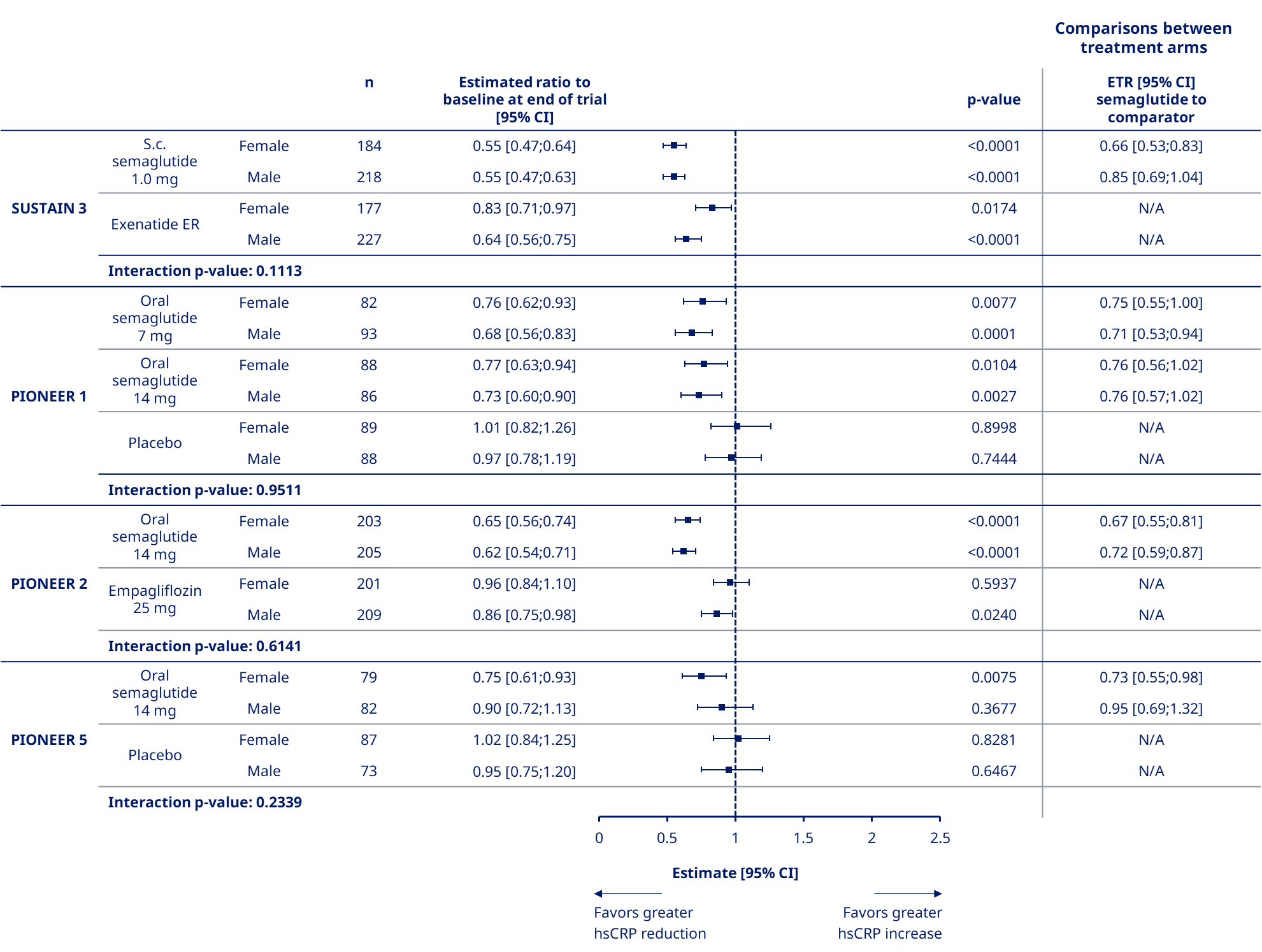
**

Ratios to baseline were analyzed using a mixed model for repeated measurements with treatment by gender as categorical fixed effects and baseline hsCRP value (log-transformed) as covariate, all nested within visit, and an unstructured residual covariance matrix on log-transformed values.
*CI* confidence interval; *ETR* estimated treatment ratio; *exenatide ER* exenatide extended release; *hsCRP* high-sensitivity C-reactive protein; *n* number of subjects with available hsCRP data; *N/A* not applicable; *s.c.* subcutaneous

**Figure S3.** Ratios to baseline at end-of-treatment for hsCRP by trial according to statin use (yes/no)


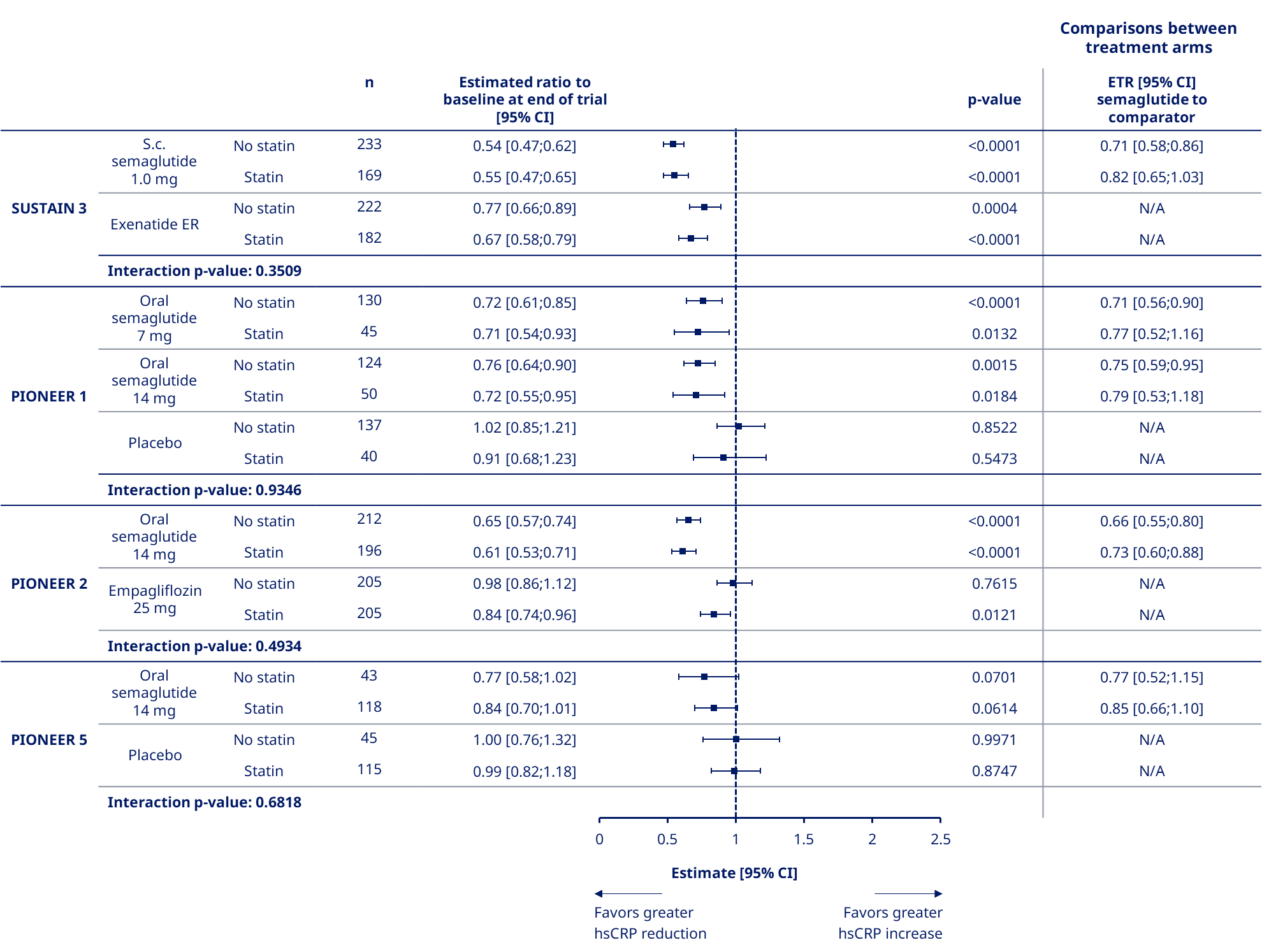


Ratios to baseline were analyzed using a mixed model for repeated measurements with treatment by baseline statin use as categorical fixed effects and baseline hsCRP value (log-transformed) as covariate, all nested within visit, and an unstructured residual covariance matrix on log-transformed values. *CI* confidence interval; *ETR* estimated treatment ratio; *exenatide ER* exenatide extended release; *hsCRP* high-sensitivity C-reactive protein; *n* number of subjects with available hsCRP data; *N/A* not applicable; *s.c.* subcutaneous

**Figure S4.** Absolute change in hsCRP from baseline to end-of-treatment by trial (semaglutide vs comparator)


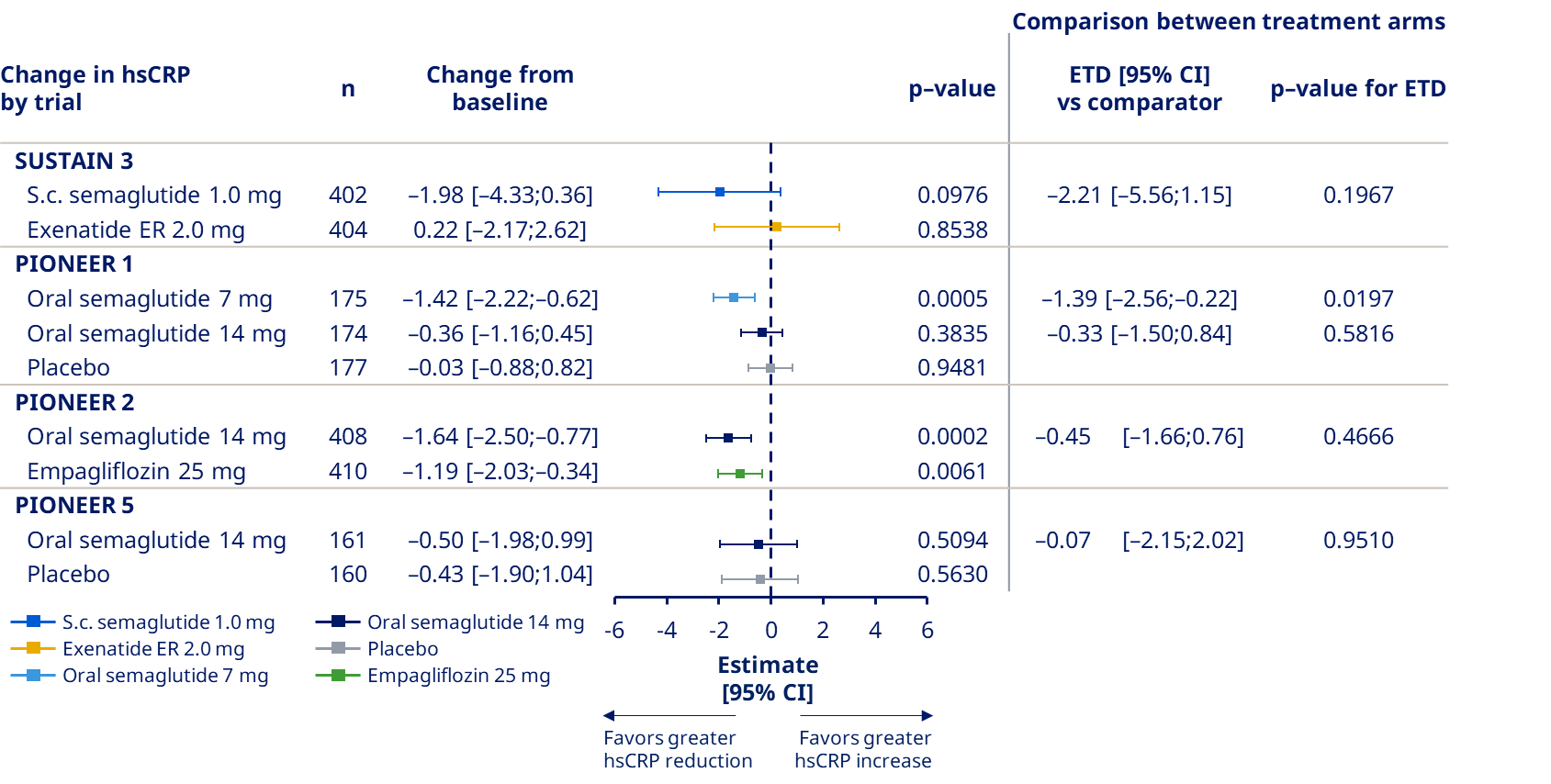


‘On-treatment without rescue medication’ data from the full analysis set. Change from baseline were analyzed using a mixed model for repeated measurements with treatment as categorical fixed effect and baseline hsCRP value as covariate, all nested within visit, and an unstructured residual covariance matrix on untransformed values. *CI* confidence interval; *ETD* estimated treatment difference; *exenatide ER* exenatide extended release; *hsCRP* high-sensitivity C-reactive protein; *s.c.*, subcutaneous

**Table S1.** Percentage of subjects who moved between hsCRP risk groups according to hsCRP category by trial – change between baseline and end of treatment

| **Study** | **Worsening in hsCRP –  Baseline hsCRP ≤3.0 mg/L  to hsCRP >3.0 mg/L at  end-of-treatment** | | **Improvement in hsCRP –**  **Baseline hsCRP >3.0 mg/L  to hsCRP ≤3.0 mg/L at  end-of-treatment** | |
| --- | --- | --- | --- | --- |
|  | **n/N** | **(%)** | **n/N** | **(%)** |
| **SUSTAIN 3** |  |  |  |  |
| S.c. semaglutide 1.0 mg | 8/212 | (3.8) | 97/190 | (51.1) |
| Exenatide ER | 22/227 | (9.7) | 68/177 | (38.4) |
| **PIONEER 1** |  |  |  |  |
| Oral semaglutide 7 mg | 10/90 | (11.1) | 31/85 | (36.5) |
| Oral semaglutide 14 mg | 7/96 | (7.3) | 17/78 | (21.8) |
| Placebo | 8/81 | (9.9) | 11/96 | (11.5) |
| **PIONEER 2** |  |  |  |  |
| Oral semaglutide 14 mg | 20/224 | (8.9) | 95/184 | (51.6) |
| Empagliflozin 25 mg | 32/214 | (15.0) | 54/196 | (27.6) |
| **PIONEER 5** |  |  |  |  |
| Oral semaglutide 14 mg | 15/80 | (18.8) | 23/81 | (28.4) |
| Placebo | 24/83 | (28.9) | 17/77 | (22.1) |

Data are n/N (%). *%* percentage of subjects; *exenatide ER* exenatide extended release; *hsCRP* high-sensitivity C-reactive protein; *n* number of subjects changing risk group; *N* full analysis set; *s.c.*, subcutaneous
